# Acral lentiginous melanoma in situ on the palm with a diffuse parallel furrow pattern

**DOI:** 10.1016/j.jdcr.2024.09.025

**Published:** 2024-10-16

**Authors:** Silas M. Money, Loretta S. Davis, Harold S. Rabinovitz, Matthew R. Powell, Kendall L. Buchanan

**Affiliations:** a Department of Dermatology, Medical College of Georgia at Augusta University, Augusta, Georgia; b Department of Pathology, Medical College of Georgia at Augusta University, Augusta, Georgia

**Keywords:** acral lentiginous melanoma, acral lentiginous melanoma in situ, dermoscopic, dermoscopy, melanoma, melanoma in situ, parallel furrow pattern, parallel ridge pattern

## Clinical presentation

**Fig 1:**
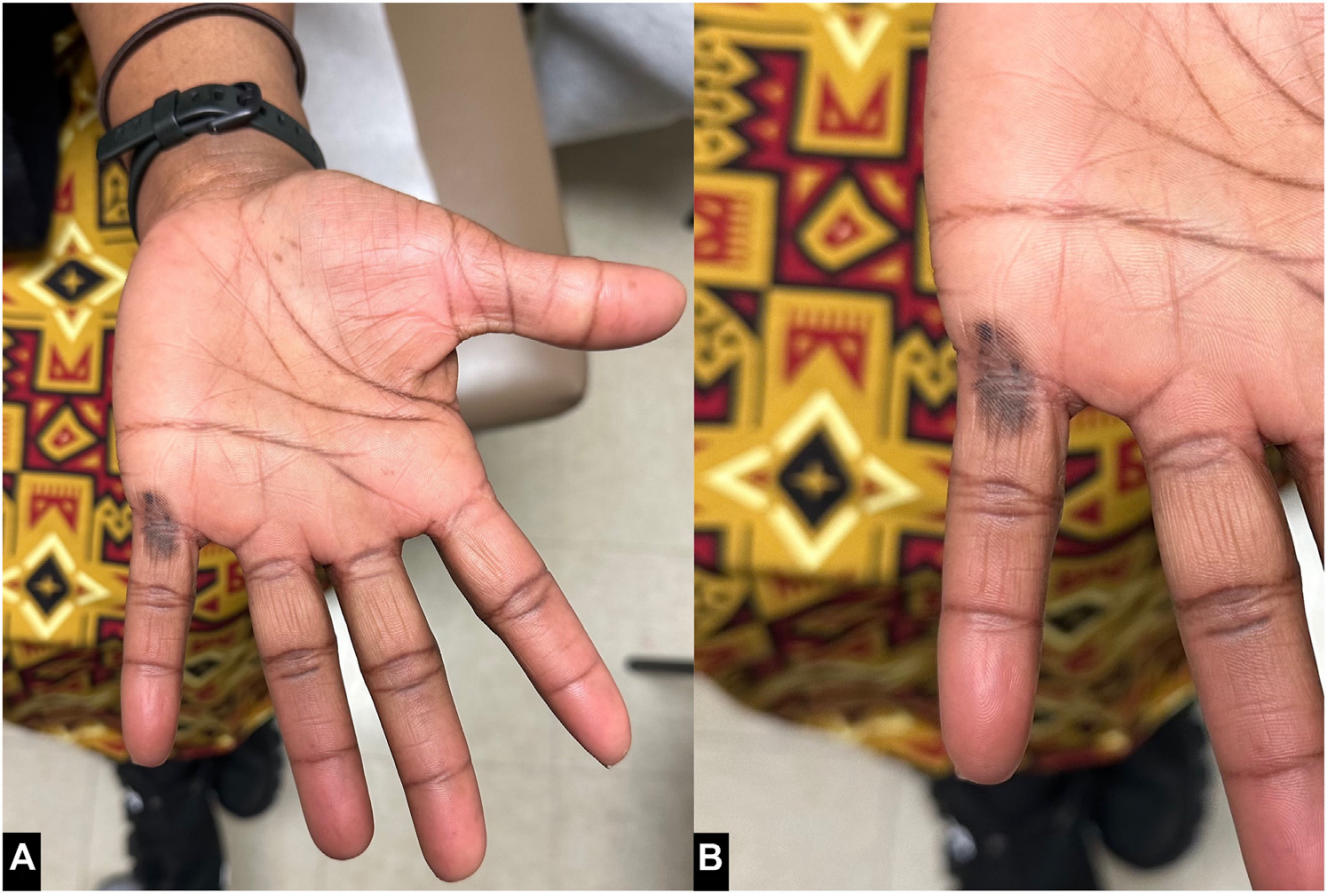
Acral lentiginous melanoma in situ, clinical appearance. A 1.4 cm × 0.7 cm ovoid patch with multiple shades of *brown*, including an eccentric blotch, on the palmar crease of the left fifth digit (**A** and **B**).

A 47-year-old Black woman reported a concerning discoloration on her left palm present for several years with recent enlargement and darkening. (Fig 1).

## Dermoscopic appearance

**Fig 2:**
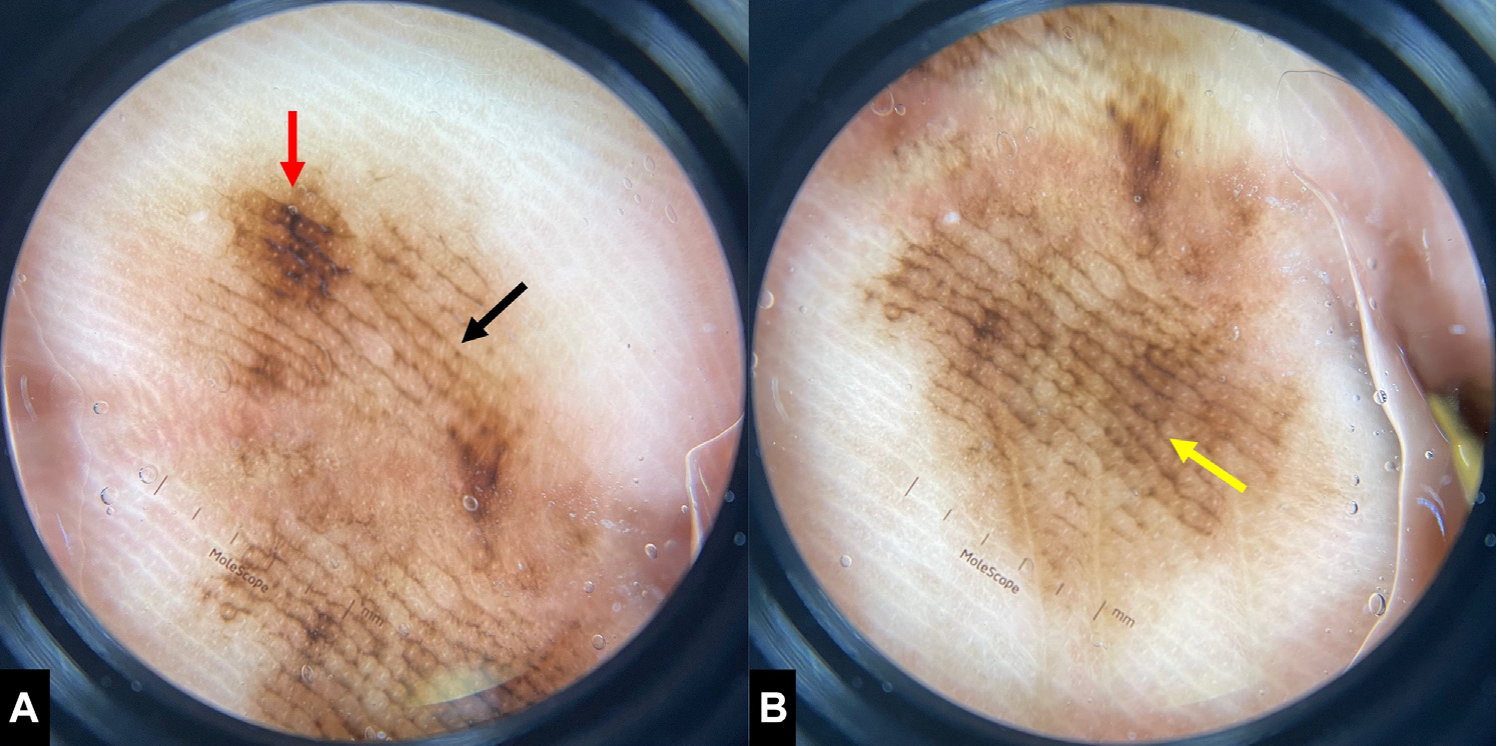
Acral lentiginous melanoma in situ, dermoscopic appearance. Polarized dermoscopy demonstrated a diffuse PFP (>90% of **A** and **B**, *black arrow*), a focal lattice-like pattern proximally (superior aspect of (**A**), *red arrow*) and a focal fibrillar pattern distally (central aspect of (**B**), *yellow arrow*), along with several shades of *brown*.

Polarized dermoscopy demonstrated a diffuse (>90%) parallel furrow pattern, a focal lattice-like pattern proximally, and a focal fibrillar pattern distally, along with several shades of brown (Fig 2).

## Histologic diagnosis

**Fig 3:**
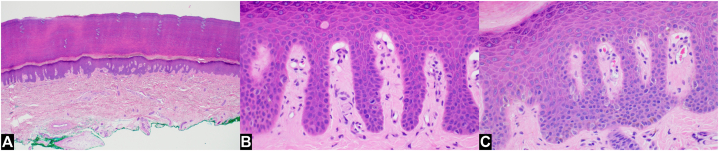
Acral lentiginous melanoma in situ, histopathologic appearance. Normal appearance of palmar acral skin at low power (**A**). Increased single unit atypical melanocytes (**B** and **C**) with 3 mitoses in a high-power field (**C**). (**A** to **C**, hematoxylin-eosin stain; original magnifications: **A,** 40×; **B** and **C,** 400×).

**Fig 4:**
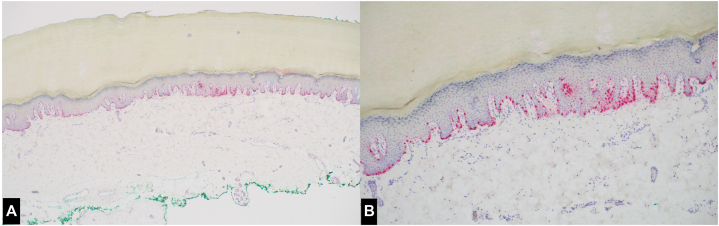
Acral lentiginous melanoma in situ, histopathologic appearance. Increased single unit atypical melanocytes with prominent dendritic processes (**A** and **B**, MelanA stain; original magnification: 40× (**A**), 100× (**B**)).

Histopathologic examination showed increased single unit atypical melanocytes with prominent dendritic processes and increased mitoses, confirming the diagnosis of acral lentiginous melanoma in situ (Figs 3 and 4).

**Key message**

Although the parallel furrow pattern is the major benign dermoscopic pattern seen in acral nevi, a diffuse parallel furrow pattern has previously been described in plantar acral lentiginous melanoma in situ.^1^ Despite this typically reassuring finding, concern was heightened by additional dermoscopic features including multiple shades of brown and an overall multicomponent pattern which included a focal fibrillar pattern. Given the absence of mechanical pressure that produces this pattern on the soles, any fibrillar pattern on the palms should be viewed with caution.^2^ Additional risk factors included the patient’s Fitzpatrick V skin type, age (borderline, > 50 is high-risk), and lesion size (>7 mm).^1^ In summary, pigmented acral lesions with predominantly benign dermoscopic patterns should be approached carefully in the setting of other suspicious dermoscopic findings, suspicious clinical findings, and well-recognized risk factors. Acral lesions are best managed by incorporating both clinical and dermoscopic findings in the context of patient risk factors to avoid missing a malignancy.

## Conflicts of interest

None disclosed.
